# Beyond the Skin: Chylous Pleural Effusion Complicating HIV-Associated Kaposi Sarcoma

**DOI:** 10.7759/cureus.110840

**Published:** 2026-06-14

**Authors:** Dina Abdelhamid, Hina Arsh, Muhammad Ali Lak, Ali Assaker, Shahzaib Khan

**Affiliations:** 1 Internal Medicine, Queens Hospital Center, Jamaica, USA; 2 Internal Medicine, Icahn School of Medicine at Mount Sinai, New York, USA; 3 Internal Medicine, Combined Military Hospital, Lahore, PAK

**Keywords:** bilateral pleural effusion, herpes virus type 8 (hhv-8), hiv aids, hiv-positive, kaposi sarcoma infiltrating thoracic duct, kaposi sarcoma (ks), malignant chylothorax, pegylated-liposomal doxorubicin, pleuropulmonary kaposi sarcoma, tumor lysis syndrome kaposi sarcoma

## Abstract

Kaposi sarcoma (KS) is an HHV-8-mediated vascular malignancy encompassing four distinct clinical variants: classic, endemic, iatrogenic, and AIDS-associated. While classically indolent, KS can pursue an aggressive course in the context of HIV infection. Pleural involvement, particularly chylous effusion, represents a rare but grave manifestation of the disease. In cases of chylous effusion, pleural fluid analysis typically reveals a triglyceride concentration >110 mg/dL or the presence of chylomicrons, findings that signify true chylous involvement. We report the case of a 33-year-old man with AIDS-associated KS complicated by chylous pleural effusion. The patient was initiated on antiretroviral therapy and received a single dose of chemotherapy, after which he developed biochemical evidence of tumor lysis syndrome 16 days later, leading to fatal clinical deterioration. This case underscores the catastrophic potential of KS complicated by chylous pleural effusion and highlights the importance of vigilance for life-threatening complications during therapy.

## Introduction

Kaposi sarcoma (KS) is an angioproliferative neoplasm driven by infection with human herpesvirus-8 (HHV-8) and occurs in four distinct epidemiologic forms: classic, endemic, iatrogenic, and AIDS-associated [1]. Among these, AIDS-related KS remains the most aggressive variant, frequently presenting with widespread cutaneous disease and visceral involvement in the setting of advanced immunosuppression [1]. Despite the widespread use of combination antiretroviral therapy, KS continues to be an important cause of morbidity and mortality among people living with HIV, particularly in those with late presentation to care [1,2].

Pleuropulmonary involvement is a well-recognized manifestation of AIDS-associated KS and is most commonly characterized by large, bilateral, hemorrhagic, or serosanguineous pleural effusions arising from highly vascular visceral pleural lesions [2]. Less commonly, KS may present with chylous pleural effusion, a rare and ominous complication resulting from disruption or obstruction of lymphatic flow, most often involving the thoracic duct [3]. Chylothorax in this context is associated with profound protein and lipid loss, severe malnutrition, immunologic compromise, and poor clinical outcomes [4]. Reported survival in patients with KS-associated chylothorax is often limited to weeks to months, reflecting advanced disease burden at the time of diagnosis [3].

The pathophysiology of KS-associated chylothorax remains incompletely understood and is thought to involve lymphatic obstruction by mediastinal nodal disease, direct pleural or thoracic duct involvement, or in situ lymphatic KS proliferation [3,5]. Diagnosis is frequently challenging, as the hemorrhagic nature of KS-related effusions may obscure the classic milky appearance of chyle, necessitating biochemical confirmation through pleural fluid triglyceride analysis [2,4]. Management is similarly complex and requires a multidisciplinary approach incorporating antiretroviral therapy, systemic chemotherapy, pleural drainage strategies, and aggressive nutritional support [2,4,6].

## Case presentation

**Figure 1:**
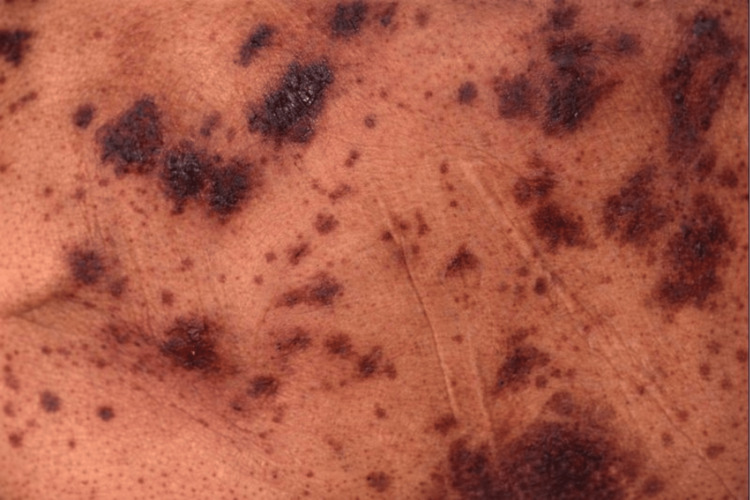
Purple violaceous skin rash over the back

A 33-year-old man with recently diagnosed HIV infection, syphilis, and KS presented to the emergency department with two weeks of progressive dyspnea on exertion, orthopnea, and worsening bilateral lower extremity swelling. He also reported poor oral intake and unintentional weight loss over the same period. He had moved to the United States in 2011 and has been living in Las Vegas in an overcrowded facility with >100 other individuals and poor living conditions. He developed multiple untreated infections and, in April, noticed a new diffuse violaceous rash over his skin (Figure 1). During his evaluation, he was diagnosed with HIV, syphilis, and KS and was treated for pneumonia. A right-sided chest tube was placed for a large pleural effusion of unclear etiology, and he was discharged home with the chest tube in situ. After discharge, he noted continuous drainage of approximately 1 L per day of pink, milky fluid from the chest tube. He subsequently moved to New York City to live with his sister, where his dyspnea and leg swelling progressively worsened over two weeks, prompting an ED visit.

On arrival, he was tachypneic but maintained oxygen saturation in the mid-90s on room air. He appeared chronically ill and cachectic with generalized anasarca. Physical examination was notable for extensive violaceous papules and plaques on the trunk and extremities, marked bilateral pitting edema to the thighs, decreased breath sounds at the right lung base with an indwelling right chest tube in place, and dullness to percussion over the lower lung fields bilaterally. Cardiovascular and abdominal examinations were otherwise unremarkable aside from distension related to fluid overload. There were no focal neurological deficits. He reported a history of sexual activity with three male partners and past use of pre-exposure prophylaxis (PrEP), though he was inconsistent with barrier protection. He denied a significant medical history prior to this year. A prior label of “penicillin allergy” had been applied after he developed rash and throat tightness during attempted benzathine penicillin treatment for syphilis two weeks earlier.

**Figure 2:**
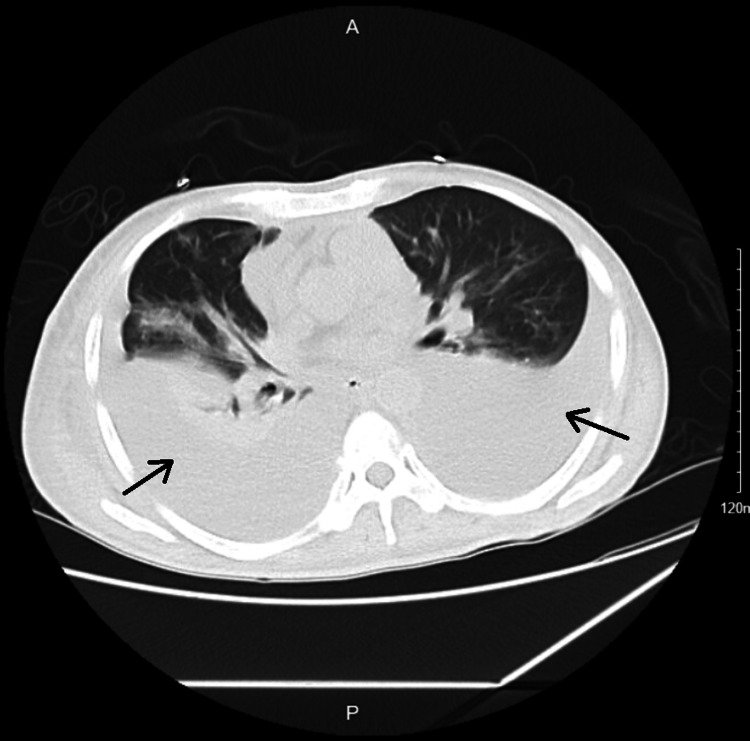
Computed tomography scan of the chest demonstrating bilateral pleural effusions (arrows)

Initial laboratory testing revealed severe hypoalbuminemia (albumin: 1.6 g/dL), hyponatremia, and evidence of advanced HIV infection with a CD4 count of 50 cells/µL (Table 1). A chest radiograph demonstrated a large right-sided pleural effusion and a moderate left-sided effusion (Figure 2). CT imaging of the chest, abdomen, and pelvis showed bilateral pleural effusions, multiple lesions in the liver and possibly spleen and spine, and a suspicious rectal mass, all concerning for disseminated KS versus a second primary malignancy. MRI of the brain revealed a small enhancing focus in the left corona radiata, and MRI of the orbits showed bilateral orbital lesions and paranasal sinus disease. Bilateral lower extremity venous duplex ultrasound was performed in view of his recent prolonged car travel and was negative for deep venous thrombosis.

**Table 1:** T cell subset

Component	Value	Reference range
ABS CD3	879 cells/mcL	861-1,947 cells/mcL
% CD3	75%	58-81%
ABS CD4	50 cells/mcL	503-1,144 cells/mcL
% CD4	4%	31-51%
ABS CD8	784 cells/mcL	279-758 cells/mcL
%CD8	67%	19-32%
4/8 Ratio	0.06 ratio	1.05-2.21 ratio
% CD4+ CD8+	0.28%	0.11-1.75%
Absolute CD4+ CD8+	3.29	2.05-37.20 /cu mm
% CD4- CD8-	3.62%	0.23-4.84%
ABS CD4- CD8-	42.24	4.15-76.64 /cu mm

The indwelling right chest tube placed in Las Vegas was connected to compatible drainage bags brought by his family, allowing bedside drainage of a large volume of pleural fluid. The effusion appeared pink and milky. Pleural fluid analysis was consistent with an exudate, and subsequent triglyceride measurement demonstrated markedly elevated levels to 324 mg/dL, establishing the diagnosis of chylothorax in the setting of KS (Table 2). A punch biopsy of cutaneous lesions was performed at the bedside and later confirmed KS with immunohistochemical positivity for CD34, CD31, D2-40, and HHV-8 and negative F8 staining, supporting the diagnosis of AIDS-associated KS with lymphatic involvement. Over the first days of admission, he continued to have severe bilateral leg edema (4+) and significant ongoing pleural drainage, but albumin supplementation increased his serum albumin modestly from 1.6 to 1.9 g/dL (Tables 3, 4), and intravenous diuretics (furosemide 40 mg in the morning and 20 mg in the afternoon) reduced edema to 3+ on the right and 2+ on the left.

**Table 2:** Pleural fluid studies

Component	Value	Reference/Interpretative range
Albumin, fluid	0.7 g/dL	Transudate if serum-effusion albumin gradient (SEAG) >1.2. Exudate if SEAG ≤1.2
Amylase, fluid	49 U/L	30-110 U/L
Cholesterol, fluid	33 mg/dL	<200 mg/dL
Glucose, body fluid	108 mg/dL	Similar to the serum range
LDH, fluid	64 U/L	Transudate if the fluid/serum LDH ratio <0.6. Exudate if fluid/serum LDH ratio >0.6
pH, fluid	7.8	Normal: 7.60–7.64
Protein, fluid	2.4 g/dL	Transudate if the fluid/serum protein ratio <0.5. Exudate if the fluid/serum protein ratio >0.5
TG, fluid	342 mg/dL	Chylothorax unlikely: <50 mg/dL. Chylothorax likely: >110 mg/dL. Equivocal: 50-110 mg/dL
Cell count, body fluid		
Appearance	Cloudy	Normal: Clear
Color, fluid	Pink	Normal: Straw-colored
WBCs	132 cells/µL	<1,000 cells/µL
RBCs	13,000 cells/µL	<100 cell/ µL
Segmented neutrophil	0%	0%
Band	0%	0%
Lymphocyte	93%	23%
Monocyte	7%	75%
Eosinophil	0%	0%
Basophil	0%	0%
Culture, body fluid	Negative	No growth
Fungal culture, body fluid	Negative	No growth

**Table 3:** Hepatic function tests ALT: alanine transaminase; AST: aspartate aminotransferase; ALK PHOS: alkaline phosphatase

Component	Value	Reference range
Albumin	1.9 g/dL	3.5-5.2 g/dL
Total protein	5 g/dL	6.6-8.7 g/dL
Total bilirubin	0.3 mg/dL	0-1.2 mg/dL
Direct bilirubin	0.1 mg/dL	0-0.2 mg/dL
ALK PHOS	104 U/L	40-129 U/L
ALT	18 U/L	0-41 U/L
AST	28 U/L	5-40 U/L
Indirect bilirubin	0.2 mg/dL	0-1 mg/dL

**Table 4:** Lipid panel LDL: low-density lipoprotein; HDL: high-density lipoprotein

Component	Value	Reference range
Cholesterol	75 mg/dL	<= 199 mg/dL
HDL cholesterol	8 mg/dL	>= 41 mg/dL
Triglyceride	307 mg/dL	<= 149 mg/dL
LDL cholesterol calculated	22 mg/dL	<= 99 mg/dL
Non-HDL cholesterol calculation	67 mg/dL	<= 129 mg/dL

The patient was evaluated by infectious disease specialists, hematology-oncology specialists, pulmonologists, and gastroenterologists. Antiretroviral therapy with oral bictegravir/emtricitabine/tenofovir alafenamide (Biktarvy) and *Pneumocystis jirovecii *prophylaxis with trimethoprim-sulfamethoxazole were initiated. Pulmonologists recommended controlled drainage of the effusion with concurrent albumin replacement. Hematology-oncology specialists planned inpatient chemotherapy with liposomal doxorubicin and placement of a venous access device once active tuberculosis could be excluded. Gastroenterologists scheduled a colonoscopy to evaluate the rectal mass and to distinguish rectal adenocarcinoma from gastrointestinal KS once airborne isolation was lifted. Quantiferon-TB testing and serial AFB cultures were negative (Table 5), allowing removal of airborne precautions and progression of the diagnostic workup. Additional infectious testing revealed low-level detectable cytomegalovirus viremia with negative Blastomyces serologies (Table 6).

**Table 5:** Tuberculosis testing AFS: acid-fast stains

AFS	No acid-fast bacilli seen by fluorochrome stain
Preliminary	No acid-fast bacilli isolated at 5 weeks
Final	No acid-fast bacilli isolated after 6 weeks

**Table 6:** CMV and blastomyces testing CMV: cytomegalovirus; PCR: polymerase chain reaction

Cytomegalovirus PCR	Detected <34.5 Abnormal	
CMV PCR Log	Detected <1.54 Abnormal	
Blastomyces antibodies	Negative	Negative: <1:1

Given the history of severe reaction to penicillin, the patient was transferred to a step-down unit (SDU) for an oral amoxicillin-graded challenge. He tolerated the challenge without reaction, and the penicillin allergy label was removed from his record, enabling appropriate treatment of neurosyphilis if confirmed. Lumbar puncture performed earlier had shown elevated opening pressure (33 mmH₂O) with clear CSF; samples were sent for further analysis to evaluate for neurosyphilis.

On return to the medical floor, colonoscopy revealed multiple polypoid, non-obstructing masses throughout the gastrointestinal tract and a frond-like/villous mass at the anus, all biopsied for histopathologic evaluation, highly suspicious for visceral KS. Meanwhile, logistical issues arose with the ongoing drainage of his pleural effusions. The right-sided chest tube from Las Vegas had a proprietary connector incompatible with local drainage systems, and the patient no longer had a supply of compatible bags. Interventional radiology was therefore consulted to replace the existing chest tube and to place a left-sided tube to allow standardized drainage during hospitalization. Despite attempts to locate compatible drainage bags through the hospital supply chain and affiliated institutions, none were available. Pulmonologists advised against pleurodesis or decortication at that time due to the underlying KS and poor nutritional status. Instead, they recommended connecting a PleurX catheter to a Pleur-evac system to facilitate intermittent drainage every other day.

Repeat pleural fluid analysis from the newly placed tubes again demonstrated exudative effusions with very high triglyceride levels, confirming bilateral chylothoraces. Recognizing the risk of worsening malnutrition from continuous chyle loss, the medical team transitioned from continuous drainage to intermittent drainage and requested a nutrition consultation. A specialized diet emphasizing short- and medium-chain fatty acids while minimizing long-chain triglycerides was initiated to reduce lymphatic flow while maintaining caloric intake.

Further staging workup included a MUGA scan, which showed low-normal left ventricular ejection fraction (48%), and a whole-body bone scan, which revealed small focal uptake in the left posterior parietal skull and left posterior eighth rib (felt to be post-traumatic) and degenerative changes in multiple joints. Ophthalmology assessment for possible CMV retinitis found improved vision with evidence of prior anterior uveitis (extensive synechiae) and cataracts; artificial tears were started, with plans for cataract extraction and synechiolysis once medically optimized. Follow-up was arranged in the ophthalmology clinic.

Throughout hospitalization, the patient experienced significant KS-related pain and odynophagia. Palliative care team optimized pain control, transitioning him from intermittent intravenous Hydromorphone to an oral regimen of extended-release morphine every 12 hours with oral hydromorphone as needed and later to a patient-controlled analgesia pump as symptoms worsened. Suspected herpetic esophagitis was evaluated with HSV serologies and PCR, and empiric acyclovir was started; atovaquone replaced trimethoprim-sulfamethoxazole when cytopenias and renal dysfunction developed, while antiretroviral therapy with Biktarvy was continued.

A peripherally inserted central catheter (PICC) line was placed by interventional radiologists for initiating inpatient chemotherapy. The patient's course was complicated by progressive hyponatremia (nadir: ~118 mEq/L), thrombocytopenia (platelets: ~33 × 10⁹/L), and hypotension requiring midodrine administration. Chemotherapy with liposomal doxorubicin was eventually started when the patient's clinical condition stabilized, with a plan to continue every three weeks.

Sixteen days after initiation of chemotherapy, the patient developed acute kidney injury with laboratory features concerning for tumor lysis syndrome (hyperphosphatemia, hypocalcemia, hyperkalemia, and hyperuricemia). His clinical condition further deteriorated with the development of increased fatigue and laboured breathing. Intensivists were consulted for an upgrade to the intensive care unit (ICU). Nephrologists recommended dialysis via a temporary hemodialysis catheter, but the patient repeatedly declined line placement despite extensive education regarding the outcome of untreated renal failure. Conservative management with bumetanide, albumin infusions, calcitriol, sevelamer, and allopurinol was thus instituted. The patient was upgraded to the ICU, with close monitoring of electrolytes, fluid status, and respiratory symptoms. The option of emergent hemodialysis was again offered when the patient's clinical status failed to improve on conservative measures; however, the patient again declined. The patient's vasopressor requirements were increasing, and despite medical efforts and multidisciplinary management, he went into shock and passed away on the morning of his third day in the ICU.

## Discussion

KS is an angioproliferative neoplasm characterized by abnormal vascular proliferation, the development of which is linked to infection with human herpesvirus 8 (HHV-8), also referred to as KS-associated herpesvirus (KSHV). Clinically, KS is categorized into four epidemiologic variants: classic KS, typically arising in middle-aged or elderly individuals; endemic KS, comprising several forms historically observed in sub-Saharan Africa prior to the AIDS epidemic; iatrogenic KS, associated with immunosuppressive therapy, particularly in organ transplant recipients as renal allograft recipients; and AIDS-associated (epidemic) KS, which emerges in the setting of HIV-related immunodeficiency [1].

Classic KS typically presents as purplish, reddish-blue, or dark brown to black macules, nodules, or plaques on the lower extremities [1], often accompanied by lymphedema, and although classically indolent and localized, it may occasionally demonstrate rapid growth, dissemination, and significant morbidity. Diagnosis requires histopathologic confirmation via biopsy, and because mucosal, nodal, and visceral involvement is uncommon, routine radiographic or endoscopic staging is not indicated in asymptomatic individuals [1].

Pleural involvement is a frequent manifestation of AIDS-related KS, occurring in 36-60% of patients and typically following the onset of cutaneous disease, declining CD4 counts, and rising HIV viral loads [2]. Pleural effusions complicating AIDS-related KS are usually large, bilateral, and serosanguineous or hemorrhagic due to highly vascular visceral pleural lesions, with accompanying interstitial or alveolar infiltrates and mediastinal or hilar adenopathy. Although pleural fluid in this patient population is generally exudative with low nucleated cell counts, mononuclear predominance, preserved glucose levels, and nondiagnostic cytology, an increasingly recognized subset of patients develops chylous pleural effusions, in which the hemorrhagic appearance may obscure the characteristic milky quality.

Chylothorax refers to the accumulation of chyle within the pleural space. Chyle is a lipid-rich lymphatic fluid with a characteristic milky appearance that is transported by the thoracic duct from the cisterna chyli to the venous system at the left jugulosubclavian junction [3]. The thoracic duct follows a posterior mediastinal course and demonstrates significant anatomical variation, with accessory tributaries present in a substantial proportion of individuals [4].

In patients with HIV-associated KS, bilateral chylothorax may arise from several mechanisms, including direct tumour involvement of the pleura or mediastinal lymph nodes, opportunistic infections related to immunosuppression or chemotherapy, or unrelated causes. Disruption or obstruction of thoracic duct flow can result in chyle accumulation within the mediastinum and subsequent leakage into the pleural space, producing chylothorax. Clinically, chylothorax manifests as dyspnea and chest discomfort [3].

The diagnosis of chylothorax is suggested by drainage of milky pleural fluid and confirmed by pleural fluid analysis demonstrating triglyceride levels >110 mg/dL (342 mg/dL in this case), cholesterol levels <200 mg/dL, pleural fluid-to-serum triglycerides ratio >1, and a pleural fluid-to-serum cholesterol ratio <1 [5].

KS is among the most common causes of pleural effusion in patients with AIDS [3]. Histopathological diagnosis of pleural KS depends on characteristic architectural features rather than cytology, as pleural fluid - typically serosanguineous or haemorrhagic - rarely contains diagnostic cells. Because KS predominantly affects the visceral pleura, closed pleural biopsy is frequently non-diagnostic, and thoracoscopy is often required, revealing characteristic cherry-red to violaceous lesions on the visceral pleural surface [3]. In many cases, clinical findings and bronchoscopic appearances support a presumptive diagnosis without tissue confirmation.

Median survival following diagnosis of pleuropulmonary KS is poor, with reported survival ranging from weeks to months and an average of approximately four months after the onset of pleural disease [3].

The precise pathogenesis of chylothorax in advanced HIV-associated KS remains incompletely understood. Most reported cases suggest lymphatic obstruction due to mediastinal nodal involvement or extensive pulmonary KS [3].

Unlike other malignancy-related pleural effusions, which are often caused by obstruction of parietal pleural lymphatics, KS-related effusions are unlikely to arise from this mechanism due to sparing of the parietal pleura. Instead, vascular endothelial growth factor (VEGF)-mediated angiogenesis and increased microvascular permeability may contribute to effusion formation. Chylothorax occurs in approximately 2% of KS-related pleural effusions, suggesting thoracic duct involvement. While this was traditionally attributed to metastatic obstruction, recent immunohistochemical evidence demonstrating HHV-8, CD34, and D2-40 co-expression, as is demonstrated in this case, supports the possibility of in situ KS development within the thoracic duct as an alternative mechanism [3,5].

Management of KS depends on disease extent and symptom burden: patients with limited, asymptomatic lesions may be observed, with compression therapy used to alleviate mild edema, whereas those with symptomatic or cosmetically distressing localized lesions are generally treated with local modalities, such as radiation therapy, excision, cryotherapy, laser ablation, or intralesional or topical agents. Systemic therapy is reserved for symptomatic visceral or mucosal disease, diffuse or bulky cutaneous involvement not amenable to localized radiation, or moderate to severe lymphedema refractory to compression. For patients requiring systemic therapy, pegylated liposomal doxorubicin is preferred in the absence of cardiac contraindications, with paclitaxel offered as an alternative for those unable to receive anthracyclines; pomalidomide may be considered for more limited disease. Subsequent-line options for progressive disease include taxanes, oral etoposide, vinca alkaloids (with or without bleomycin), vinorelbine, gemcitabine, or pomalidomide, with treatment individualized based on performance status, comorbidities, and patient preferences [1]. Investigational strategies under evaluation include mTOR inhibitors, targeted antiangiogenic therapies, and immune checkpoint inhibitors [2].

Chylothorax management relies on pleural drainage, pleurodesis, and/or shunting, alongside systemic chemotherapy and highly active antiretroviral therapy (HAART). Adjunctive supportive care emphasises dietary modification, including the use of low-fat or medium-chain triglyceride (MCT) diets, supplementation of essential fatty acids and fat-soluble vitamins, and additional protein intake when chyle output is substantial. Long-chain triglycerides (LCTs) undergo digestion to monoglycerides and free fatty acids and are transported as chylomicrons through the intestinal lymphatic channels. By contrast, MCTs are absorbed directly into the portal venous system without lymphatic involvement. Reducing dietary LCT intake, therefore, decreases chyle formation [6].

## Conclusions

This case underscores the fulminant and fatal potential of AIDS-associated KS complicated by bilateral high-output chylous pleural effusions. Despite antiretroviral and chemotherapeutic intervention, rapid deterioration ensued, complicated by tumor lysis syndrome. This report adds to the limited literature on KS-associated chylothorax and emphasizes the importance of early recognition, multidisciplinary management, and further investigation to improve outcomes in patients with advanced disease.

## References

[1] Krown SE, Singh JC (2026). Classic Kaposi sarcoma: clinical features, staging, diagnosis, and treatment. UpToDate.

[2] Vempilly JJ (2026). Pleural effusions in HIV-infected patients. UpToDate.

[3] Verma K, Haverkamp M, Kayembe M, Musimar Z (2013). Chylothorax associated with non-endemic Kaposi’s sarcoma. S Afr J HIV Med.

[4] Feller-Kopman D, Maldonado F (2021). Pleural disease. Clin Chest Med.

[5] Pantanowitz L, Dezube BJ (2008). Kaposi sarcoma in unusual locations. BMC Cancer.

[6] Cherian S, Umerah OM, Tufail M, Panchal RK (2019). Chylothorax in a patient with HIV-related Kaposi's sarcoma. BMJ Case Rep.

